# Inhibition of heparan sulfate synthesis by resveratrol in mucopolysaccharidosis type I and IIIB

**DOI:** 10.1007/s11011-026-01999-2

**Published:** 2026-10-10

**Authors:** Estera Rintz, Aneta Szulc, Urszula Maliszewska, Grzegorz Węgrzyn

**Affiliations:** Department of Molecular Biology, University of Gdansk, Wita Stwosza 59, 80-308 Gdansk, Poland

**Keywords:** Lysosomal storage diseases, Glycosaminoglycans, Cellular models, Substrate reduction therapy

## Abstract

Mucopolysaccharidoses (MPS) are a group of pediatric disorders that are distinguished based on a lysosomal enzyme deficit leading to accumulation of glycosaminoglycans (GAGs). One of the GAGs is heparan sulfate (HS) which accumulates in cells inside the body, but the most devastating symptoms are caused by its accumulation in the central nervous system. Resveratrol, a natural compound, reduces the HS accumulation in both MPS I and MPS IIIB. In this study, we investigated the effects of resveratrol on HS metabolism in cellular models of MPS I and MPS IIIB, with particular focus on its role in GAG synthesis and degradation. Resveratrol treatment resulted in a consistent reduction of HS levels in both models, as confirmed by dot-blot analysis. To determine the underlying mechanism, we analyzed the expression of genes involved in GAG biosynthesis and turnover, as well as heparanase activity. The expression of key genes involved in the early stages of GAG biosynthesis was modulated by resveratrol, suggesting inhibition of substrate production. In contrast, expressions of genes associated with extracellular matrix remodeling and GAG turnover were largely unchanged in MPS I and only partially affected in MPS IIIB. Analysis of the heparanase enzyme activity showed no significant changes in either model. Although alterations in extracellular heparanase activity were observed in MPS IIIB cells, these changes were normalized rather than enhanced by resveratrol treatment. Our results indicate that resveratrol reduces HS accumulation through inhibition of synthesis rather than induction of unspecific degradation by heparanase, supporting its potential as a substrate-reduction strategy in MPS.

## Introduction

Mucopolysaccharidoses (MPS) are a group of inherited disorders distinguished by a deficiency in the activity of one of the lysosomal enzymes (Rintz et al. 2024a; Nagpal et al. 2022). Mutations in genes encoding lysosomal enzymes involved in the degradation of glycosaminoglycans (GAG) cause accumulation of these compounds, leading to devastating symptoms. The most severe ones include neurological complications such as developmental delay, hyperactivity, sleep disorders, and speech difficulties. Nevertheless, MPS are systemic diseases that affect the entire organism, causing organomegaly, movement problems, growth delays, cardiorespiratory failure, and much more. Depending on the MPS type (classified according to the type of affected gene and the kind of accumulated GAG(s)), symptoms may differ in type or severity. Most patients survive to the second decade of life.

Current MPS treatment includes enzyme replacement therapy (ERT) and hematopoietic stem cell transplantation (HSCT). While these therapies can be successful in managing systemic symptoms, they are not effective for brain disorders. Thus, patients with MPS I may receive ERT or HSCT as they experience more systemic symptoms, while in the case of MPS III, with a primary neurological component, no therapy is currently available. Other drawbacks of ERT include its high costs and constant need for frequent hospitalization, while HSCT reveals some efficacy only when applied before the 2nd year of age, with additional limitations connected to finding a suitable donor, graft versus host disease, and complications connected to the procedure (Rintz et al. 2024a; Nagpal et al. 2022).

In this work, we focused on two MPS types, MPS I and MPS IIIB, in which heparan sulfate (HS) accumulates. HS is stored in virtually all cells throughout the body, but its most devastating symptoms result from its accumulation in the central nervous system (CNS). HS also activates the immune response in the brain of patients, making their symptoms even more severe (Muenzer et al. 2024). In our previous studies, we found that resveratrol (Rintz et al. 2020), a natural compound, is able to reduce HS accumulation in MPS IIIB (Rintz et al. 2023, 2026) and MPS I (Rintz et al. 2024b). Resveratrol activates autophagy in MPS models through multiple molecular pathways, including mTOR inhibition and activation of transcription factors such as TFEB and FOXO3 (Rintz et al. 2026). This observation is consistent with reports showing that stimulating autophagy can enhance the clearance of stored substrates in lysosomal storage disorders (Settembre et al. 2013). In addition, we observed that resveratrol treatment led to an increase in α-L-iduronidase (IDUA) enzymatic activity in MPS I cells together with stimulation of lysosomal receptor – M6PR, suggesting that its beneficial effects may not be limited to substrate degradation alone, but could also involve improved lysosomal function and enzyme trafficking (Rintz et al. 2024b). Our latest study showed that resveratrol also reduces inflammation in both peripheral nerves and CNS of MPS IIIB mice. A connection between autophagy and immune response was also demonstrated as a potential pathogenesis of HS accumulation (Rintz et al. 2026). Moreover, resveratrol, when combined with ERT, increased the efficacy of the therapy by increasing enzyme activity and reducing HS (Rintz et al. 2024b). Interestingly, even though the MPS IIIB mouse model is a knock-out in *NAGLU* (thus, mice do not have any detectable NAGLU enzyme activity), resveratrol was still able to decrease HS accumulation (Rintz et al. 2026). Here, using cellular models of MPS I and MPS IIIB, we aimed to test whether resveratrol inhibits HS synthesis and/or activates other lysosomal enzyme(s) that are non-specific for HS reduction.

## Materials and methods

### Cells and cell cultures

Fibroblast cell lines were obtained from patients suffering from MPS type I (GM00798; deficient alpha-L-iduronidase; homozygous for a c.1293G > A substitution in exon 9 of the *IDUA* gene p.W402X), IIIB (GM00156; deficient N-acetyl-alpha-D glucosaminidase activity; homozygous for a c.1876 C > T transition in the *NAGLU* gene p.R626X), and the control line (GM00969; healthy control). These lines were purchased from the Coriell Institute for Medical Research (Camden, NJ, USA). Cells were cultured under standard conditions (37 °C, DMEM medium supplemented with 10% fetal bovine serum, 95% humidity, 5% CO2, 1% pen-strep antibiotic). Each experiment was performed in three independent biological replicates.

### Enzyme and resveratrol

Aldurazyme (laronidase, recombinant human α-L-iduronidase) was from Sanofi/Genzyme (Cambridge, MA, USA) and used at a final concentration of 0.58 mg/mL. Resveratrol (Trans-3,4′,5-Trihydroxystilbene; 1,3-Benzenediol, 5-[2-(4-hydroxyphenyl)ethenyl]-) was purchased from Santa Cruz Biotechnology (Cat#sc-215997, Dallas, Texas, USA) and used at a final concentration of 20, 80 or 160 µM. All compounds were added to cell cultures and incubated for 24 h.

### Heparan sulfate accumulation analysis

In cellular experiments, fibroblasts (5 × 10^5^ cells) were plated in 10 cm plates and incubated overnight. The cells were treated with resveratrol, an enzyme, or both and then incubated for 24 h. Cells were then lysed in the lysis buffer (1% Triton X-100, 0.5 mM EDTA, 150 mM NaCl, 50 mM Tris, pH 7.5), supplemented with a mixture of protease and phosphatase inhibitors (Roche Applied Science, Penzberg, Germany). The protein lysate was obtained by centrifugation, and protein concentration was measured using a BCA kit according to the manufacturer’s instruction (Cat#23225, ThermoFisher, Waltham, Massachusetts, USA). For dot-blotting analysis of HS, 50 µg of protein was used with Heparan Sulfate Proteoglycan 2 antibody (A74) (Cat# NBP2-23523, Novus Biologicals, Centennial, Colorado, USA), diluted 1:500. As a secondary antibody, goat anti-mouse antibody (Cat#31431, ThermoFisher, Waltham, Massachusetts, USA), diluted 1:4000 was employed. Protein detection was performed using a Bio-Rad apparatus. Briefly, 30 µL of cell lysate was loaded into each well and transferred onto a PVDF membrane, which had been previously activated in methanol for 5 min. The membrane was then blocked with milk for 2 h, followed by incubation with the appropriate primary and secondary antibodies. Protein signals were detected in a darkroom using X-ray film. The resulting signals were quantified using ImageJ software.

### Radiolabelled GAG incorporation assay

Measurement of radiolabelled GAGs was conducted as previously described (Moskot et al. 2015). MPS I and MPS IIIB fibroblasts were seeded into 6-well plates at 1.5 × 10^5^ cells/well in DMEM (Cat#11995-065, ThermoFisher, Waltham, Massachusetts, USA) containing 10% FBS (Cat#A5256701, ThermoFisher, Waltham, Massachusetts, USA) and 1 × Antibiotic-Antimycotic (Cat#15240062.00, ThermoFisher, Waltham, Massachusetts, USA). Upon reaching ~ 80% confluence (~ 24 h), cells were treated with resveratrol (0, 20, 80 or 160 µM) for 3 h. Medium was then replaced with glucose/pyruvate-free DMEM containing supplements (10% FBS and 1 × Antibiotic-Antimycotic) plus D-[1-³H]-glucosamine-HCl (10 µCi/mL; American radiolabelled Chemicals, ART 0292 A). After 24 h incubation, medium was removed, and cells were washed 6 times with DPBS (cat#14190144), followed by papain digestion (3 h, 60 °C) in the papain buffer (0.5% papain in 200 mM), phosphate buffer (Na₂HPO₄-NaH₂PO₄, pH 6.4; 100 mM sodium acetate, 10 mM Na₂EDTA, 5 mM L-cysteine). Following digestion, 50 µL aliquots were spotted onto filter paper, transferred to scintillation vials with 2 mL EcoLite(+) (MP Biomedicals, Cat#882475), and ³H incorporation was quantified using a MicroBeta 2 counter (PerkinElmer). Radiolabeled GAG levels were normalized to DNA content. Aliquots of 10 µL were used for DNA measurement with Quant-iT™ PicoGreen™ dsDNA Assay Kit (Cat#P7589). For GAG precipitation, remaining digests were supplemented with 50 µL of 1% CPC and 25 µg chondroitin sulfate (carrier), incubated overnight at 4 °C, and centrifuged (15 min, 14,000 rpm). Pellets were resuspended in 500 µL 3 M NaCl, followed by addition of 1.5 mL 80% ethanol/0.5% sodium acetate, re-incubation at 4 °C, and re-centrifugation. Final pellets were dissolved in 100 µL of ddH₂O for scintillation counting, as described above.

### Gene expression analysis

Total RNA was isolated from cultured cells, and 2 × 10^5^ cells were plated into 6-well plates. After overnight incubation, cells were treated with either resveratrol, enzyme, or both. Following 24 h incubation, cells were homogenized and lysed with a QIAshredder column (Cat#79656, Qiagen, Venlo, The Netherlands), with the use of a solution containing guanidine isothiocyanate and β-mercaptoethanol. RNA extraction was performed using the RNeasy Mini kit (Cat#74106, Qiagen, Venlo, The Netherlands) and treatedt with Turbo DNase (Cat#AM1907, Invitrogen ™ Thermo Fisher Scientific, Waltham, Massachusetts, USA), according to the manufacturers’ instructions. The quality of isolated RNA samples was assessed by NanoDrop.

Reverse transcription quantitative real-time PCR (RT-qPCR) was performed to determine the expression fold change levels of the selected genes. A total amount of 300 ng RNA was reverse transcribed using the NG dART RT Kit (Cat#E0801-02, EURx, Gdansk, Poland). RT-qPCR analyses were conducted using a LightCycler 480 II system (Roche, Basel, Switzerland) with SYBR Green Master Mix (Roche, Basel, Switzerland). The primers used are listed in Table 1. Gene expression efficiency was calculated using the 2^−ΔΔCT^ method, with *GAPDH* serving as the reference gene.

**Table 1:** Primers used for RT-qPCR

Gene name	Primers sequence	
*IDUA*	Forward	5’- CAGGAGATACATCGGTAGGTACG − 3’
	Reverse	5’- TCATGGAGACGTTGTCAAAGTC − 3’
*NAGLU*	Forward	5’- ACCGCTATTACCAGAATGTGTG − 3’
	Reverse	5’- CCATCCAGTCTATCTCTCGCTC − 3’
*XYLT1*	Forward	5’- CACACCCAAGTCCGCTCATC − 3’
	Reverse	5’- GTTCGCACTTTCTCTTTCGGC − 3’
*EXT1*	Forward	5’- GCTCTTGTCTCGCCCTTTTGT − 3’
	Reverse	5’- GTGGTGCAAGCCATTCCTAC − 3’
*EXTL2*	Forward	5’- CTTCCTGGGAGAGTAATGGGG − 3’
	Reverse	5’- CCTACGCAACATGAGCATCTTG − 3’
*HS2ST1*	Forward	5’- GCTCCTCAGGATTATGATGCC − 3’
	Reverse	5’- TTTCTCGGACTTCGTGTCTTG − 3’
*HYAL3*	Forward	5’- AAGAACCAACTCGGCCTCTAT − 3’
	Reverse	5’- TGTGGTGGATCTGGTAGGCA − 3’
*HSPG2*	Forward	5’- ATGGCCGGGCAGTAAATGG − 3’
	Reverse	5’- GCTGGCTCTGGAATAAATCCG − 3’
*GAPDH*	Forward	5’- GGAGCGAGATCCCTCCAAAAT − 3’
	Reverse	5’- GGCTGTTGTCATACTTCTCATGG − 3’

### Immunoblotting

Control, MPS I, and MPS IIIB cell lines (6 × 10^5 cells) were seeded onto 10-cm plates and incubated overnight to reach approximately 80% confluency. The following day, cells were treated with 80 µM resveratrol and incubated for 24 h. Cells were then collected by trypsinization and centrifuged at 300 × g for 10 min. Cell pellets were lysed in lysis buffer supplemented with protease and phosphatase inhibitors (Sigma-Aldrich, St. Louis, MO, USA) and centrifuged at 12,000 rpm for 30 min at 4 °C. The supernatants were collected, and protein concentrations were determined using a BCA assay (ThermoFisher, Waltham, Massachusetts, USA). Proteins were analyzed using the Wes automated capillary electrophoresis system (Simple Western, ProteinSimple, San Jose, CA, USA) according to the manufacturer’s instructions. Protein separation was performed using the 12–230 kDa Separation Module (#SM-W003) with 4 µg of protein per sample. Detection was performed using either the Anti-Mouse Detection Module (#DM-002) or the Anti-Rabbit Detection Module (#DM-001), according to the manufacturer’s protocol. The following primary antibodies were used at a 1:50 dilution: anti-EXTL2 (Cat. #PA5-30024, Invitrogen, ThermoFisher, Waltham, Massachusetts, USA), anti-XYLT1 (Cat. #PA5-67627, Invitrogen, ThermoFisher, Waltham, Massachusetts, USA), and anti-EXT1 (Cat. #PA5-106907, Invitrogen, ThermoFisher, Waltham, Massachusetts, USA). Total protein, measured using the Total Protein Detection Module for chemiluminescence (#DM-TP01, ProteinSimple, San Jose, CA, USA), was used as the loading control. Protein levels were quantified using ImageJ by measuring band intensities. The intensity of each target protein was normalized to the corresponding total protein signal and expressed as fold change relative to the control.

### Heparanase enzyme assay

Fibroblasts were seeded into 10-cm dishes (5 × 10⁵ cells/plate) and incubated overnight. Cells were then cultured in a serum-free medium and treated with resveratrol, enzyme, or both for 24 h, depending on the experimental group. Conditioned media were collected, and cells were washed with cold PBS and lysed in the heparanase assay buffer supplemented with protease inhibitors. Samples were homogenized, centrifuged at maximum speed (20 min, 4 °C), and supernatants were collected. Protein concentration was determined using the BCA assay, and samples were adjusted to 10–30 µg protein per well. Heparanase activity was measured using a commercial ELISA-based kit, according to the manufacturer’s instructions (Cat#Ra001-PCE-K, Amsbio, Oxfordshire, UK). Briefly, samples were added in duplicates and incubated at 37 °C for 1 h. Collected media was applied directly into the well without additional steps. After washing, streptavidin-HRP was added, followed by substrate development. The reaction was stopped with 0.12 M HCl, and absorbance was measured at 450 nm. Enzymatic activity was calculated from optical density values, normalized to protein concentration, and expressed as ng of HS degraded per minute per mg of protein. Enzymatic activity in the culture medium was calculated according to the manufacturer’s instructions and expressed as the amount of HS degraded per minute.

### Statistical analysis

Mean values ± standard deviation (SD) are used to present results of experiments. GraphPad 9.0.0 software was used for statistical analyses. The Shapiro-Wilk test and the Levene test were used for checking the normality of the distribution of variables and the homogeneity of the variances, respectively. When data were not normally distributed the Kruskal-Wallis and Dunn tests were used for analysis. Otherwise, ANOVA and Tukey’s post hoc tests were performed. Differences were considered statistically significant when *p* < 0.05.

## Results and discussion

**Fig. 1:**
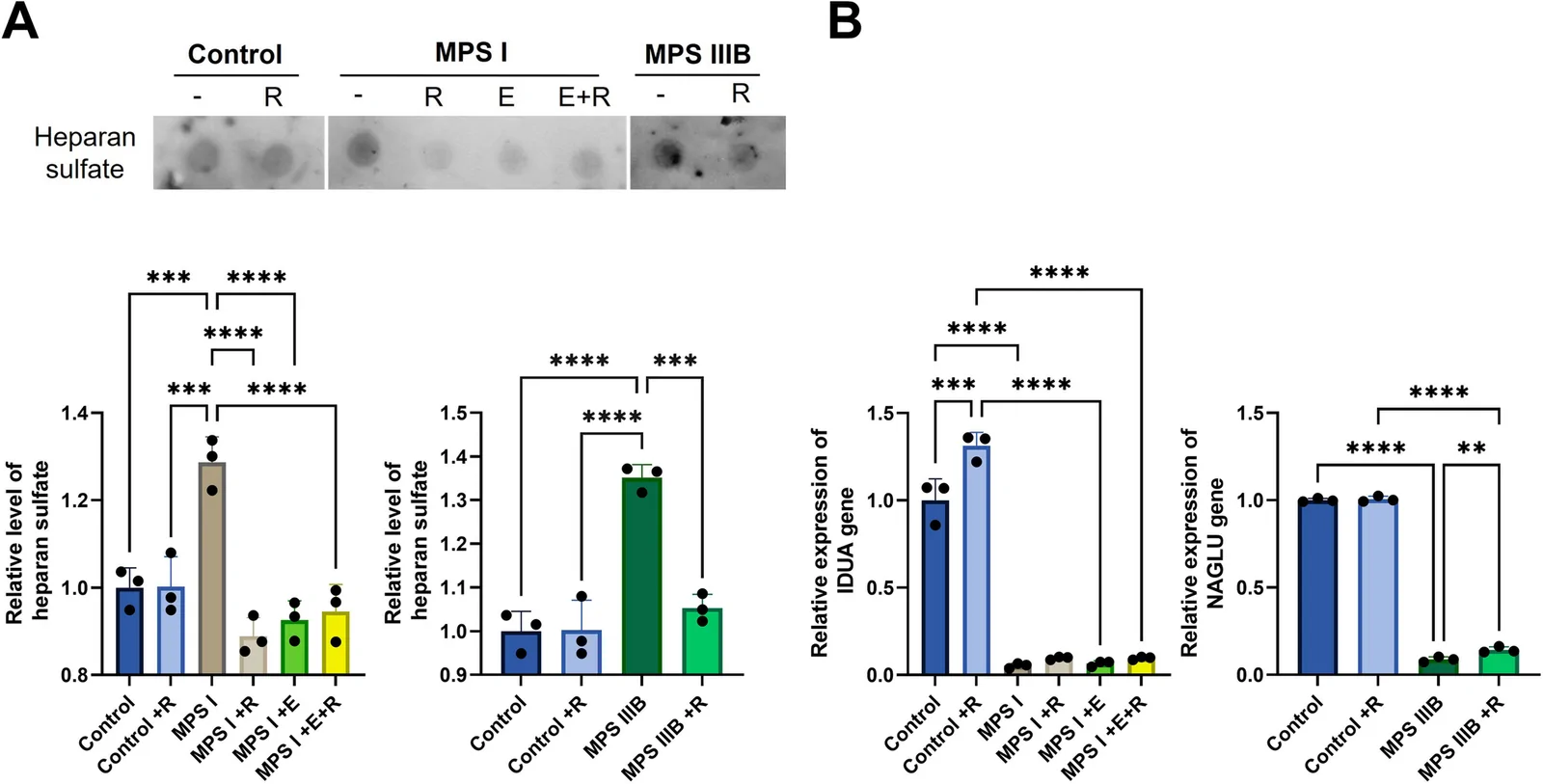
Resveratrol reduces HS accumulation in MPS I and IIIB. (**A**) Representative dot blot of HS quantification with statistical analysis for both MPS I and IIIB cells treated with enzyme (E), resveratrol 80 µM (R), or both (E + R). (**B**) Expression of the *IDUA and NAGLU* genes in MPS I and MPS IIIB cells, respectively, relative to the control cell line, was assessed by RT-qPCR and calculated using the double delta Ct method. Data are presented as mean ± SD (N = 3). Significant differences are indicated as follows: **, p < 0.01; ***, p < 0.001; ****, p < 0.0001

In this study, we confirmed previous findings that resveratrol reduces levels of HS in MPS I and MPS IIIB cells (Fig. 1A), and sought to understand better the molecular mechanisms underlying the action of this polyphenolic compound. *IDUA* and *NAGLU* genes’ expressions were also analyzed following resveratrol treatment. We observed an increase in *NAGLU* expression in MPS IIIB cells after resveratrol treatment (Fig. 1B).

**Fig. 2:**
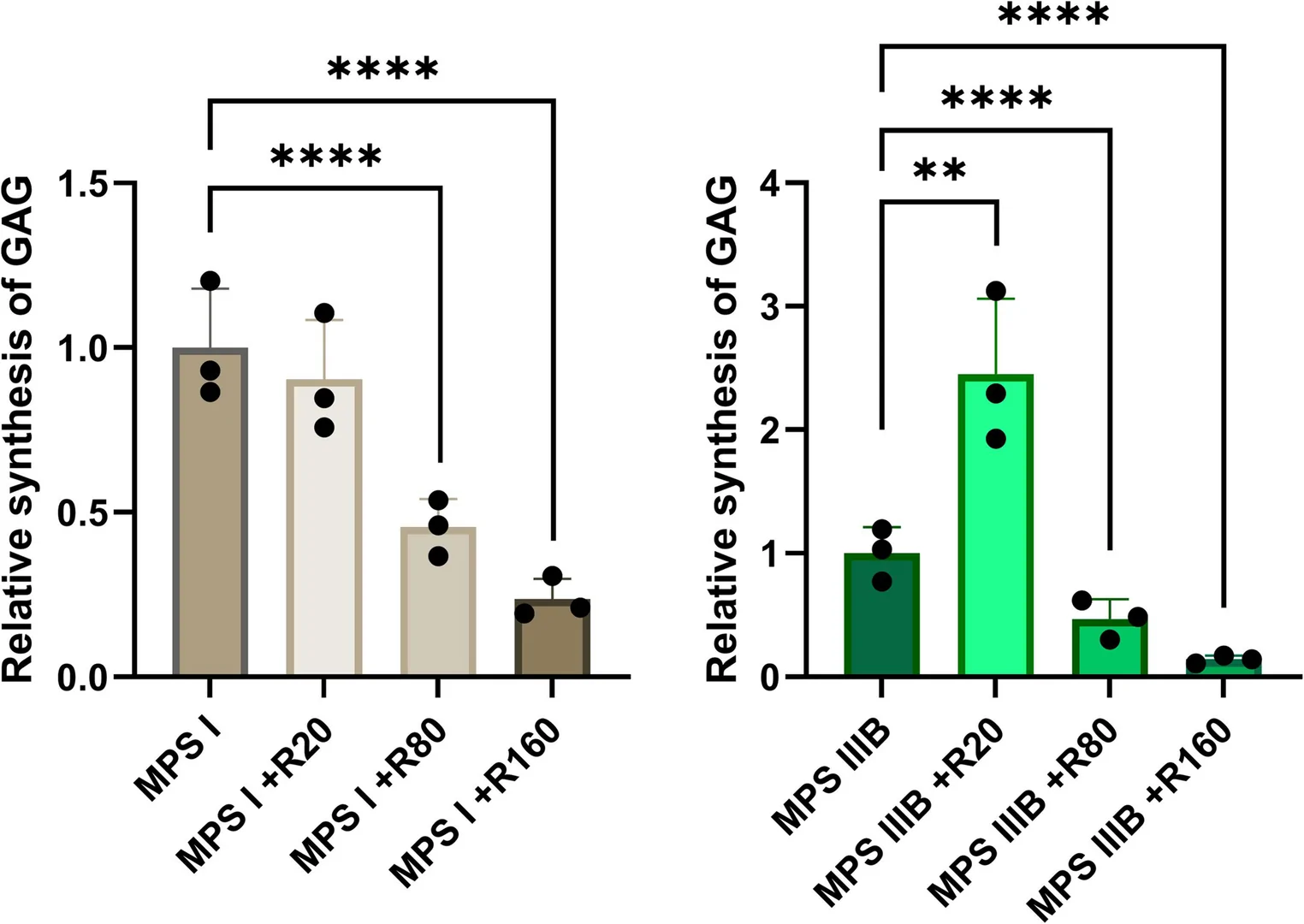
Resveratrol inhibits synthesis of GAGs in both MPS I and IIIB. The relative GAG synthesis in MPS I and IIIB cell lines treated with increased resveratrol concentration (20, 80 and 160 µM) is shown. Data are presented as mean ± SD (N = 3). Significant differences are indicated as follows: **, p < 0.01; ****, p < 0.0001

Given that the reduction in GAG levels could result not only from enhanced degradation but also from decreased synthesis, we next investigated whether resveratrol affects GAG production. To address this, we measured GAG synthesis by metabolic incorporation of labelled precursors into newly synthesized GAGs. Our results show that resveratrol significantly inhibited GAG synthesis in both MPS I and MPS IIIB cells in a dose-response mode (Fig. 2). Interestingly, in MPS IIIB cells, 20 µM resveratrol significantly increased GAG synthesis, suggesting a concentration-dependent, biphasic response. The mechanism underlying this effect remains unclear and requires further investigation. Overall, this finding suggests that resveratrol might be considered as a potential multifunctional molecule for substrate reduction therapy. However, the extent to which the observed effect specifically reflects reduced HS synthesis remains to be determined, as the incorporation of the radiolabeled compound into total GAGs was measured, which does not specifically distinguish HS from other GAG species.

To further investigate the mechanisms by which resveratrol inhibits HS synthesis, we analyzed the expression of genes encoding proteins involved in the GAG biosynthesis and assembly of proteoglycans. Based on our previous studies with resveratrol, we selected 80 µM for further experiments, as this concentration has been shown to effectively modulate cellular processes, including autophagy and immune responses, without affecting cell viability (Rintz et al. 2023, 2026). Key genes involved in biosynthesis of HS at different stages, like initiation of synthesis of GAG linker region (xylosyltransferase 1, encoded by *XYLT1*) and elongation of HS chain (exostosin-like glycosyltransferase 2, encoded by *EXTL2*, exostosin glycosyltransferase 1, encoded by *EXT1*), were examined. Moreover, the expression of *HS2ST1*, coding for heparan sulfate 2-O-sulfotransferase 1, which catalyzes HS sulfation, was assessed.

**Fig. 3:**
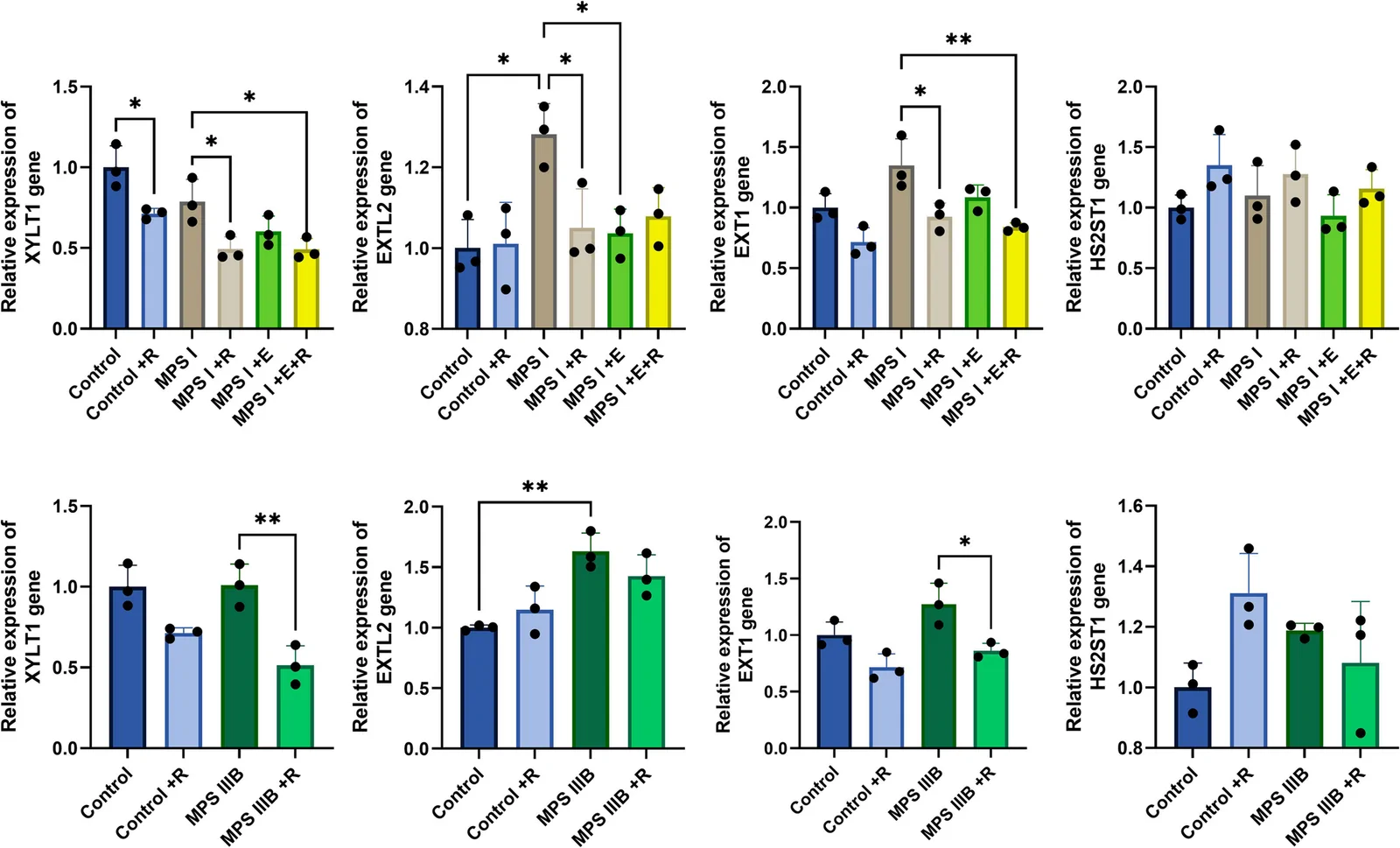
Resveratrol regulates expression of genes coding for enzymes involved in HS synthesis. Expression of *XYLT1*,* EXTL2*,* EXT1*, and *HS2ST1* genes in MPS I and IIIB cells, relative to the control cell line, treated with enzyme (E), resveratrol 80 µM (R), or both (E + R), was assessed by RT-qPCR and calculated using the double delta Ct method. Data are presented as mean ± SD (*N* = 3). Significant differences are indicated as follows: *, *p* < 0.05; **, *p* < 0.01

Previous studies have shown that silencing of *XYLT1* leads to inhibition of GAG synthesis in MPS III cells (Dziedzic et al. 2010). We found that resveratrol inhibited HS synthesis at an early stage though decreasing *XYLT1* expression in both MPS I and MPS IIIB. Moreover, when combined administration of resveratrol and recombinant a-L-iduronidase was applied, the *XYLT1* expression was also lower in MPS I (Fig. 3).

**Fig. 4:**
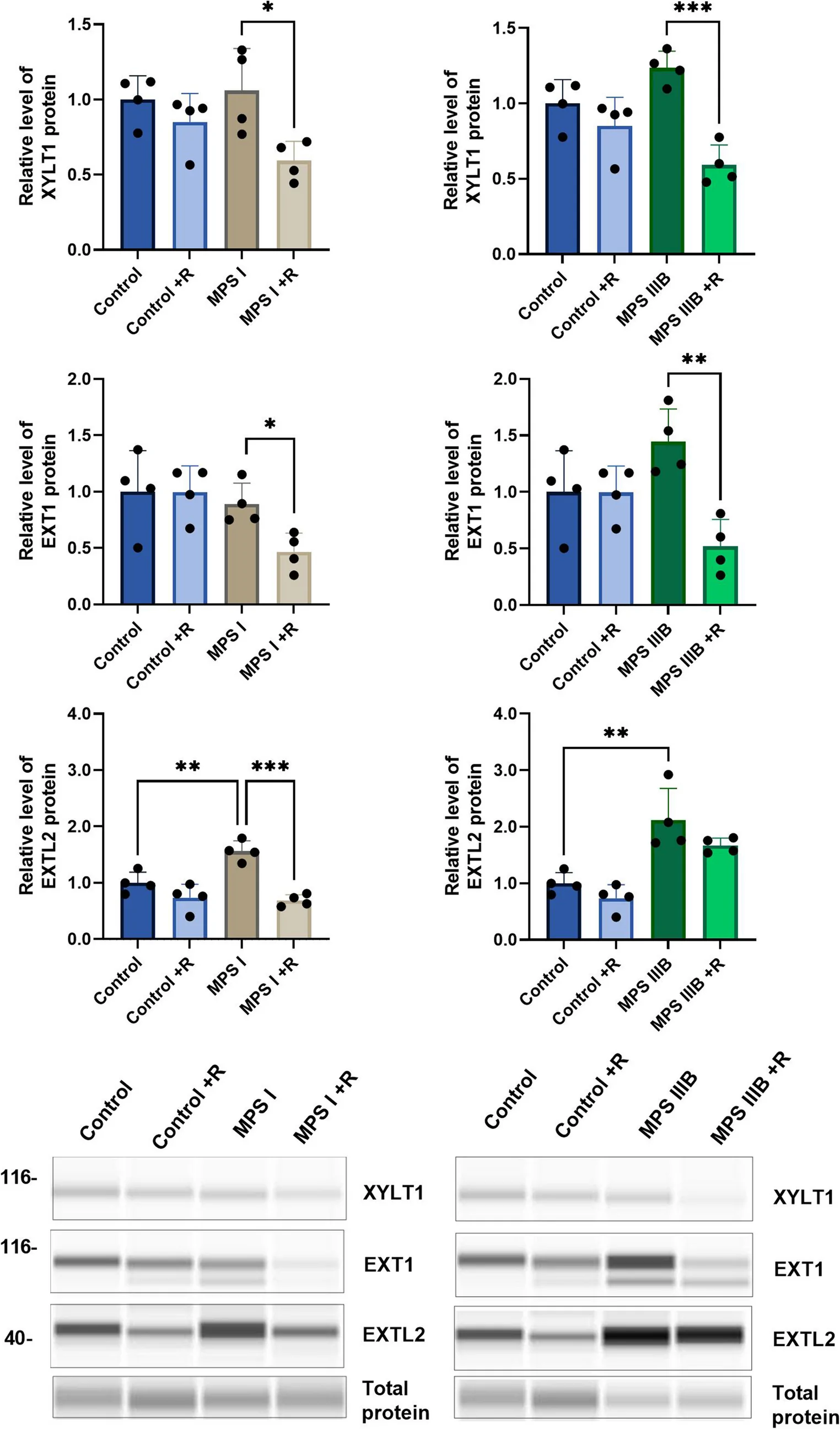
Resveratrol regulates the level of proteins involved in HS synthesis. Relative protein levels of XYLT1, EXT1, and EXTL2 shown as fold change to control. MPS I and IIIB cells were treated with resveratrol 80 µM (R). Representative western blots in the lower panel. Data are presented as mean ± SD (*N* = 4). Significant differences are indicated as follows: *, *p* < 0.05; **, *p* < 0.01; ***, *p* < 0.001

*EXTL* genes play important roles in the HS chain elongation process. We found that resveratrol decreased the expression of *EXTL2* in MPS I but not in MPS IIIB cells (Fig. 3). In fact, the expression of *EXTL2* was increased in both MPS I and MPS IIIB in comparison to healthy controls (Fig. 3). Importantly, the expression patterns of *EXTL2*, *EXT1*, and *XYLT1* at the gene level were consistent with their corresponding protein levels (Fig. 4), further supporting these observations. The increased *EXTL2* expression might be related to the HS accumulation, as studies in MPS III models have shown that silencing of *EXTL2* resulted in reduced lysosomal storage in MPS IIIA and IIIC cell lines (Kaidonis et al. 2010; Canals et al. 2015), which is consistent with our results (Fig. 1). Importantly, targeting different steps of the GAG biosynthetic pathway using RNAi consistently resulted in decreased substrate production, with combined silencing approaches showing even greater effects than single-gene expression inhibition (Dziedzic et al. 2012). Moreover, these strategies have been shown to reduce GAG accumulation without significant cytotoxicity in most models (Chmielarz et al. 2012).

*EXT1* expression was also decreased by resveratrol in both MPS I and MPS IIIB (Fig. 3). The *EXT1* gene encodes a key enzyme involved in HS biosynthesis, being responsible for HS chain elongation. Its importance in regulating substrate levels has been demonstrated in a genetic model for substrate reduction therapy, where partial reduction of HS synthesis through *Ext1* heterozygosity in mice led to a 30–50% decrease in substrate levels and improvement in disease pathology, including in the brain (Lamanna et al. 2012). Similarly, pharmacological downregulation of *EXT1* reduced the efficiency of early steps of GAG biosynthesis and led to decreased production of HS (Moskot et al. 2014). Since *XYLT1* acts at an early stage of the pathway, its modulation has a strong impact on overall GAG synthesis, supporting its role as an effective target for substrate reduction strategies in MPS.

We also investigated *HS2ST1*, a gene encoding the enzyme involved in the post-synthetic modification of HS by introducing 2-O sulfation. It does not affect HS production, but significantly influences its structure and biological interactions. Therefore, changes in *HS2ST1* expression may reflect alterations in HS functional properties. However, we did not find any statistically significant differences in the expression of *HS2ST1* between untreated cells and those treated with resveratrol (Fig. 3).

**Fig. 5:**
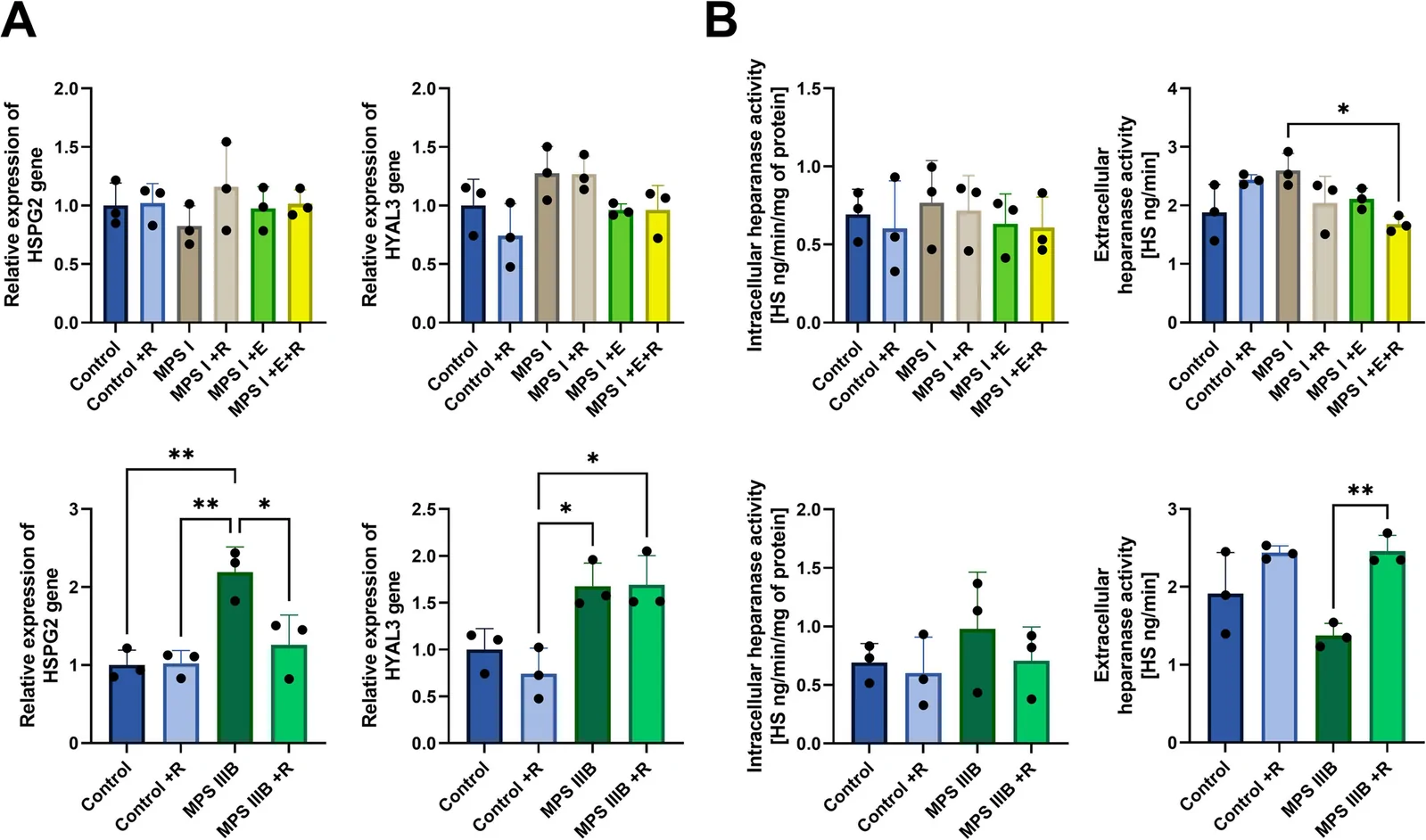
Resveratrol involvement in degradation of HS by heparanases. (**A**) Relative expression of *HSPG2* and *HYAL3* genes in MPS I and IIIB cells, relative to the control cell line, treated with enzyme (E), resveratrol 80 µM (R), or both (E + R), was assessed by RT-qPCR and calculated using the double delta Ct method. (**B**) Intra- and extra-cellular activity of heparanase in MPS I and IIIB, treated as described for panel A. Data are presented as mean ± SD (*N* = 3). Significant differences are indicated as follows: *, *p* < 0.05; **, *p* < 0.01

To more deeply analyze the functional basis of resveratrol action in MPS, we performed additional experiments to test resveratrol’s ability to stimulate HS degradation. HSPG2 and HYAL3 were selected as representative molecules to investigate whether resveratrol may also influence the extracellular environment and GAG turnover, in addition to its previously observed effects on autophagy and GAG clearance. We estimated the expression of genes related to extracellular matrix (ECM) remodeling and regulation of GAG turnover, *HSPG2* (encoding perlecan) and *HYAL3* (encoding hyaluronidase 3). We additionally assessed intra- and extra-cellular heparanase activity to determine whether resveratrol may enhance non-specific HS degradation. In MPS I cells, we did not observe any significant changes in *HSPG2* or *HYAL3* expression, and intracellular heparanase activity also remained unchanged (Fig. 5A). Interestingly, extracellular heparanase activity was reduced only after a combined treatment with resveratrol and the enzyme, suggesting that resveratrol itself does not directly enhance HS degradation in this MPS type.

In MPS IIIB cells, the results were slightly different. We observed an increased expression of *HSPG2* compared to control cells, which was normalized after resveratrol treatment, pointing to a partial correction of ECM-related alterations. *HYAL3* expression was also elevated in MPS IIIB cells, but in this case resveratrol did not have any effect in this case (Fig. 5).

When heparanase activity was analyzed, we did not detect any significant differences in intracellular activity in either model (Fig. 5B). On the contrary, extracellular heparanase activity was decreased in MPS IIIB compared to control cells and was restored to near-control levels after resveratrol treatment (Fig. 5B). This suggests that the issue is not simply related to enzyme activity, but rather to its distribution or function in the extracellular environment.

The limitations of this study should be acknowledged. The experiments were performed using a single patient-derived fibroblast line for each disease model and were limited to a single time point (24 h). Thus, the findings may not fully reflect disease-related changes in affected tissues, patient-to-patient variability, or changes over time. In addition, our gene expression analysis focused mainly on enzymes involved in HS synthesis. It did not include enzymes involved in the synthesis of other GAGs, such as chondroitin sulfate, which would also be beneficial. Future studies using additional patient-derived lines, disease-relevant cell types, and multiple time points will be needed to confirm these findings.

## Conclusion

Resveratrol reduces HS levels in both MPS I and MPS IIIB cells. Our findings support the model of inhibition of GAG biosynthesis as the predominant mechanism underlying this effect, potentially involving changes in key enzymes acting at early stages of GAG synthesis, including the products of *EXT1* and *XYLT1*. In contrast, our results provide no clear evidence for a direct effect of resveratrol on HS degradation. In MPS I, no significant changes were detected in the expression of genes related to GAG turnover or in heparanase activity. In MPS IIIB, alterations in *HSPG2* expression and extracellular heparanase activity may rather reflect normalization of the extracellular environment than activation of degradation pathways. Nevertheless, additional functional studies are required to confirm the contribution of reduced GAG biosynthesis to the observed decrease in HS levels.

## Data Availability

No datasets were generated or analysed during the current study.

## References

[CR1] Canals I, Benetó N, Cozar M, Vilageliu L, Grinberg D (2015) EXTL2 and EXTL3 inhibition with siRNAs as a promising substrate reduction therapy for Sanfilippo C syndrome. Sci Rep 5:13654. 10.1038/srep13654 PMC4561882 26347037

[CR2] Chmielarz I, Gabig-Ciminska M, Malinowska M, Banecka-Majkutewicz Z, Wegrzyn A, Jakobkiewicz-Banecka J (2012) Comparison of siRNA-mediated silencing of glycosaminoglycan synthesis genes and enzyme replacement therapy for mucopolysaccharidosis in cell culture studies. Acta Biochim Pol 59:697–702 23251908

[CR4] Dziedzic D, Wegrzyn G, Jakóbkiewicz-Banecka J (2010) Impairment of glycosaminoglycan synthesis in mucopolysaccharidosis type IIIA cells by using siRNA: a potential therapeutic approach for Sanfilippo disease. Eur J Hum Genet 18(2):200–205. 10.1038/ejhg.2009.144 PMC2987185 19690584

[CR3] Dziedzic D, Narajczyk M, Gabig-Ciminska M, Jakobkiewicz-Banecka J (2012) Simultaneous siRNA-mediated silencing of pairs of genes coding for enzymes involved in glycosaminoglycan synthesis. Acta Biochim Pol 59:293–298 22590693

[CR5] Kaidonis X, Liaw WC, Roberts AD, Ly M, Anson D, Byers S (2010) Gene silencing of EXTL2 and EXTL3 as a substrate deprivation therapy for heparan sulphate storing mucopolysaccharidoses. Eur J Hum Genet 18(2):194–199. 10.1038/ejhg.2009.143 PMC2987189 19690583

[CR6] Lamanna WC, Lawrence R, Sarrazin S, Lameda-Diaz C, Gordts PL, Moremen KW, Esko JD (2012) A genetic model of substrate reduction therapy for mucopolysaccharidosis. J Biol Chem 287(43):36283–36290. 10.1074/jbc.M112.403360 PMC3476295 22952226

[CR8] Moskot M, Montefusco S, Jakóbkiewicz-Banecka J, Mozolewski P, Węgrzyn A, Di Bernardo D, Węgrzyn G, Medina DL, Ballabio A, Gabig-Cimińska M (2014) The phytoestrogen genistein modulates lysosomal metabolism and transcription factor EB (TFEB) activation. J Biol Chem 289(24):17054–17069. 10.1074/jbc.M114.555300 PMC4059147 24770416

[CR7] Moskot M, Jakóbkiewicz-Banecka J, Kloska A, Smolińska E, Mozolewski P, Malinowska M, Rychłowski M, Banecki B, Węgrzyn G, Gabig-Cimińska M (2015) Modulation of expression of genes involved in glycosaminoglycan metabolism and lysosome biogenesis by flavonoids. Sci Rep 5:9378. 10.1038/srep09378. Erratum in: Sci Rep (2016) 6:22809. https://doi.org/10.1038/srep22809. Erratum in: Sci Rep (2016) 6:38118. https://doi.org/10.1038/srep38118 PMC4369724 25797591

[CR9] Muenzer J, Ho C, Lau H, Dant M, Fuller M, Boulos N, Dickson P, Ellinwood NM, Jones SA, Zanelli E, O’Neill C (2024) Community consensus for Heparan sulfate as a biomarker to support accelerated approval in Neuronopathic Mucopolysaccharidoses. Mol Genet Metab 142(4):108535. 10.1016/j.ymgme.2024.108535 39018614

[CR10] Nagpal R, Goyal RB, Priyadarshini K, Kashyap S, Sharma M, Sinha R, Sharma N (2022) Mucopolysaccharidosis: A broad review. Indian J Ophthalmol 70(7):2249–2261. 10.4103/ijo.IJO_425_22 PMC9426054 35791104

[CR12] Rintz E, Pierzynowska K, Podlacha M, Węgrzyn G (2020) Has resveratrol a potential for mucopolysaccharidosis treatment? Eur J Pharmacol 888:173534. 10.1016/j.ejphar.2020.173534 32877657

[CR14] Rintz E, Podlacha M, Cyske Z, Pierzynowska K, Węgrzyn G, Gaffke L (2023) Activities of (Poly)phenolic antioxidants and other natural autophagy modulators in the treatment of Sanfilippo disease: remarkable efficacy of resveratrol in cellular and animal models. Neurotherapeutics 20(1):254–271. 10.1007/s13311-022-01323-7 PMC10119361 36344724

[CR11] Rintz E, Banacki M, Ziemian M, Kobus B, Wegrzyn G (2024a) Causes of death in mucopolysaccharidoses. Mol Genet Metab 142(3):108507. 10.1016/j.ymgme.2024.108507 38815294

[CR15] Rintz E, Ziemian M, Kobus B, Gaffke L, Pierzynowska K, Wegrzyn G (2024b) Synergistic effects of resveratrol and enzyme replacement therapy in the mucopolysaccharidosis type I. Biochem Pharmacol 229:116467. 10.1016/j.bcp.2024.116467 39111602

[CR13] Rintz E, Podlacha M, Cyske Z, Pierzynowska K, Węgrzyn G, Gaffke L (2026) TFEB, FOXO3 and TLR4 in resveratrol-induced autophagy in a mucopolysaccharidosis IIIB mouse model. Exp Mol Med 58(2):436–471. 10.1038/s12276-026-01643-0. Erratum in: Exp Mol Med (2026) PMC12992813 41639426

[CR16] Settembre C, Fraldi A, Medina DL, Ballabio A (2013) Signals from the lysosome: a control centre for cellular clearance and energy metabolism. Nat Rev Mol Cell Biol 14(5):283–296. 10.1038/nrm3565 PMC4387238 23609508

